# A Case of Non-ketotic Hyperglycemic Hemichorea-Hemiballismus in a 54-Year-Old Male Individual With Type 2 Diabetes Mellitus

**DOI:** 10.7759/cureus.77820

**Published:** 2025-01-22

**Authors:** Gracie L Gelnett, Ejiofor Achalu, Jonathon Pious, Joseph Wheeler, Richard Chapman, Issac Johnson, Rebecca Newton

**Affiliations:** 1 Family Medicine, Edward Via College of Osteopathic Medicine, Auburn, USA; 2 Family Medicine, Christ Health Center, Birmingham, USA

**Keywords:** complications of diabetes mellitus, hemichorea-hemiballismus, nonketotic hyperglycemia, non-ketotic hyperglycemia hemichorea-hemiballismus syndrome, types 2 diabetes, uncontrolled hyperglycemia

## Abstract

Non-ketotic hyperglycemic hemichorea-hemiballismus syndrome is a rare neurological condition associated with hyperglycemia from uncontrolled type 2 diabetes mellitus. The disease is diagnosed via clinical presentation, laboratory evaluation, and neuroimaging. With a prompt diagnosis, glycemic control is initiated as the mainstay of treatment. Additional management of chorea symptoms can be completed with neuroleptics. Here, we report a case of a 54-year-old male individual presenting with unilateral, involuntary, and uncontrollable muscle contractions secondary to non-ketotic hyperglycemia. He was managed with strict glycemic control only. Significant improvement in symptoms was noted two months after diagnosis. A complete recovery is expected within six months of onset.

## Introduction

Non-ketotic hyperglycemic hemichorea-hemiballismus syndrome is a rare neurological condition. Often noted as a complication of uncontrolled or undiagnosed type 2 diabetes mellitus, it has been referred to as “diabetic striatopathy” (DS). DS is characterized by a triad of non-ketotic hyperglycemia, uncontrollable and irregular muscle contractions, and lesions of hyperintensity or hyperdensity in the basal ganglia either via T1-weighted magnetic resonance imaging (MRI) or computed tomography (CT), respectively [1-4]. Patients diagnosed with DS present with an average blood glucose ranging from 306-481mg/dL on admission [2,5-7]. Uncontrollable muscle contractions, noted as either chorea or ballismus based on severity and presentation, are typically unilateral, but have been reported as bilateral in a small subset of cases [2,4,8,9]. A majority of cases present with neuroimaging findings seen solely on the contralateral basal ganglia in relation to the chorea present on physical exam, with a few notable exceptions of ipsilateral or bilateral basal ganglia lesions [6,7]. The putamen is always involved in DS neuroimaging, but the caudate nucleus and globus pallidus may also be affected [2,5]. The epidemiology of this disease shows a higher frequency among elderly women of Asian descent, with a mean age onset of 71 years and a prevalence estimated at less than one in 100,000 people [5,7,10]. Additionally, it is reported more often in patients previously diagnosed with uncontrolled type 2 diabetes mellitus, but can also present in patients with undiagnosed diabetes mellitus or patients with type 1 diabetes mellitus [5].

The underlying pathophysiology of non-ketotic hyperglycemic hemichorea-hemiballismus syndrome remains unclear, but several mechanisms have been proposed. Potential etiologies include infection, hyperviscosity, ischemia, or alterations in neurotransmitter activity within the basal ganglia [4,5,7,9]. When considering how alterations in neurotransmitters can cause DS, gamma-aminobutyric acid (GABA) alterations are proposed to have a significant role. It has been hypothesized that the brain favors anaerobic metabolism as its main source of energy when the body is in a non-ketotic hyperglycemic state. The switch to anaerobic metabolism leads to the activation of the Krebs cycle and ultimately rapid depletion of GABA. The depletion of GABA leads to disinhibition of the subthalamic nucleus and basal ganglia, which contributes to hyperkinetic movements, like chorea and ballismus [5,7]. While this hypothesis is valuable, it may not explain why chorea can occur in hypoglycemic states, as well as in cases where euglycemia has been restored [5].

Recognizing and accurately diagnosing DS is crucial for prompt treatment. Once diagnosed, appropriate management of hyperglycemia can lead to significant improvement or resolution of symptoms. Consequently, the mainstay of treatment is glycemic control [1-7]. Other treatment options include neuroleptics (e.g. haloperidol) or GABA agonists (e.g. benzodiazepines) for the management of chorea symptoms [1,6]. The prognosis of DS is favorable with treatment, with most patients having complete resolution of symptoms. In a recent study conducted by Chua et al., 77% of patients managed with optimal glycemic control only had a complete recovery with a median of two days, while only 59% of patients managed with both optimal glycemic control and the use of a neuroleptic agent had a complete recovery with a median of two weeks [5]. According to a meta-analysis in 2002, nearly 95% of patients with DS receiving appropriate medication to maintain euglycemia will have a complete recovery within six months of the onset of the chorea [7]. Additionally, the patients were found to have a resolution of lesions noted on imaging. A minority of patients will progress to either partial resolution of symptoms or even future recurrence of chorea, especially if uncontrolled blood glucose is present [7].

## Case presentation

The patient is a 54-year-old Caucasian male individual with a past medical history of uncontrolled type 2 diabetes mellitus, hypertension, hyperlipidemia, thyrotoxicosis, and tobacco dependence. Five days before admission to the hospital, the patient presented to the emergency department with a primary concern of left upper and lower extremity pain. The pain initially presented in the left lower extremity only. Physical examination found no abnormalities in range of motion, strength, or sensation. The patient was further examined with a left hip and shoulder x-ray; both reported no fracture, dislocation, or other acute process present. No labs or further imaging were taken at this time. During this emergency room visit, muscle strain was diagnosed. The patient was discharged and prescribed diclofenac 50 mg for pain management and methocarbamol 500 mg for muscle relaxation.

**Figure 1:**
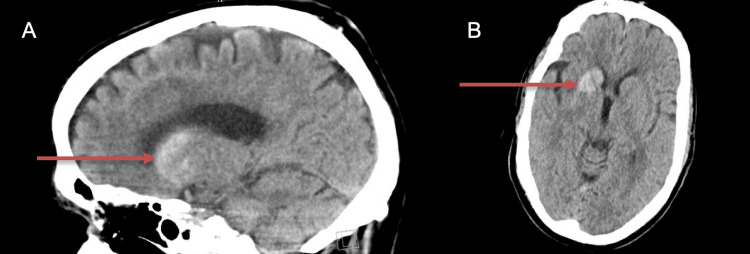
Head CT without contrast in sagittal (Panel A) and transverse views (Panel B). Noted hyperdense lesion (red arrow) in the right basal ganglia and anterior limb of the right internal capsule.

Five days later, the patient returned to the emergency department with a concern of left upper and lower extremity pain as well as left upper extremity, left lower extremity, and left face twitching that was spontaneous and uncontrollable. The patient was in no acute distress but had notable writhing and unpredictable jerking movements affecting his left side with associated left ankle pain. Physical examination found no abnormalities in range of motion, strength, or sensation. No focal deficits were noted. The cerebellar exam was normal. A urinalysis reported (+1) protein, (+4) glucose, and notably no ketones. Additionally, point-of-care glucose reported a glucose level of 422 mg/dL. Other pertinent labs reported at this time included a negative syphilis test, a negative HIV test, and normal thyroid levels. The patient was admitted to the hospital for further evaluation and neurology was consulted. Head CT without contrast was remarkable for an oval 3.1 x 2.3 x 1.5 cm mildly hyperdense lesion in the right basal ganglia and anterior limb of the right internal capsule (Figure 1).

**Figure 2:**
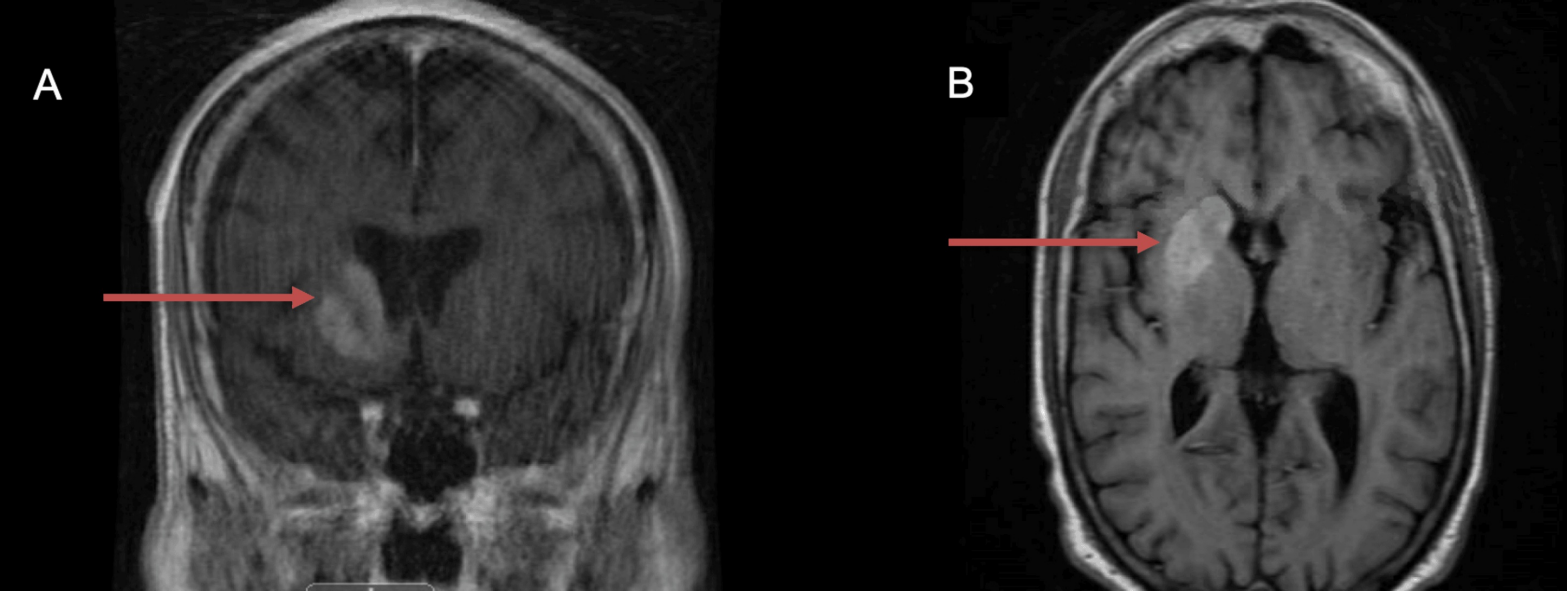
MRI of the brain in coronal (Panel A) and transverse (Panel B) views. Noted increased T1 signal and contrast enhancement (red arrow) involving the right caudate head and the right lentiform nuclei.

Additionally, a computed tomography angiography (CTA) of the head and neck was negative for any significant vascular disease. An MRI of the brain with and without contrast reported no territorial infarcts but increased T1 signal and contrast enhancement involving the right caudate head and the right lentiform nuclei (Figure 2). Additionally, the contrast enhancement was noted to be unilateral, not resulting in mass effect or midline shift.

Altogether, a physical exam, laboratory studies, and neuroimaging helped determine the diagnosis of hemichorea-hemiballismus syndrome. The diagnosis was likely secondary to hyperglycemia from uncontrolled type 2 diabetes mellitus, as our patient was inconsistent with his diabetes care. Our patient had uncontrolled type 2 diabetes mellitus for about a six-year duration. He admitted to noncompliance of medications even though he was prescribed to take 25 units of insulin glargine (Lantus, Sanofi, Paris, France) every day along with a weekly Ozempic .25 injection (Novo Nordisk A/S, Bagsværd, Denmark). Additionally, the patient’s last hemoglobin A1C was greater than 15% just one month prior to diagnosis. This patient remained admitted to the hospital for two days. During this time, the patient had blood glucose levels ranging from 115-358 mg/dL. While inpatient, the team's primary goals were symptomatic management of the patient's spasticity, chorea, and pain; improving blood glucose control; and continuing management of his other chronic diseases. During his admission, the patient was managed with acetaminophen 650 mg and baclofen 10 mg for muscle relaxation and associated pain as needed. For glycemic control, the patient was managed on his home medication insulin regimen of insulin aspart injection (NovoLog, Novo Nordisk A/S, Bagsværd, Denmark) with meals and insulin glargine 25 units per day. Additionally, all home medications were continued for the management of chronic diseases. On the day of discharge, his blood glucose was 115 mg/dL, showing enormous improvement from admission hemoglobin A1C of 13.8%. The patient was educated on the importance of compliance and continuation of glycemic control with his regimen. His pain was managed with acetaminophen and baclofen on discharge.

After discharge, the patient was closely followed in an outpatient clinic. During his one-week follow-up visit, our patient was noted to have continued significant chorea that affected his daily activities. The patient was prescribed semaglutide .25 mg injection weekly, insulin glargine 34 units daily, and metformin 500 mg twice a day to continue glycemic management. With this regimen, his blood glucose ranged between 200-340 mg/dL. With minimal improvement in his symptoms four weeks after discharge, strict glycemic control was continued with Ozempic .5 mg injection weekly, Lantus 34 units daily, and metformin 1,000mg twice a day. With this regimen, his blood glucose improved to between 140-270 mg/dL. Finally, eight weeks after discharge, the patient had incomplete but notable improvement of chorea. The patient's current diabetic regimen includes Ozempic 1 mg injection per week, Lantus insulin injection 34 units per day, metformin 1000 mg twice a day, and empagliflozin (Jardiance, Boehringer Ingelheim Pharmaceuticals, Inc., Ridgefield, CT, USA) 10 mg per day. Now, the patient admits to blood glucose readings between 110-160 mg/dL. Additionally, the patient's hemoglobin A1C has decreased from above 15% to 8.4% in three months due to compliance with a strict medication regimen. The patient will continue being monitored outpatient and work on fine motor skills with physical and occupational therapists to increase the likelihood of a full recovery within six months.

## Discussion

Non-ketotic hyperglycemia hemichorea-hemiballismus syndrome or “diabetic striatopathy” (DS) is a rare complication most often seen in uncontrolled diabetes mellitus. DS can be diagnosed with the triad of hyperglycemia, uncontrollable muscle contractions known as chorea or ballismus, and hyperintensity or hyperdensity of basal ganglia on MRI or CT, respectively [1-4]. While the pathophysiology is not fully understood, several etiologies have been proposed, including hyperviscosity, ischemia, and alterations in neurotransmitter activity within the basal ganglia [4,5,7,9]. Currently, the mainstay of treatment involves glycemic control. While the addition of neuroleptics for the management of chorea has been reported in several studies, treatment with glycemic control-only has been documented to have a higher likelihood of complete recovery of DS with a shorter median recovery time. Based on two studies with limited subjects, treatment with glycemic control-only has been shown to lead to complete resolution of DS in 77% of patients within a median of two days, while treatment with both glycemic control and a neuroleptic agent contributes to complete resolution of DS in 59% of patients with a median of two weeks [5].

Our patient, a 54-year-old Caucasian male individual with uncontrolled type 2 diabetes mellitus, presented with unilateral pain and uncontrollable muscle contractions on the left side of his body. Pertinent labs showed an elevated blood glucose of 422 mg/dL, negative syphilis testing, negative HIV testing, and thyroid levels that were normal. At this time, imaging was completed and neurology was consulted. The non-contrast CT of his head showed a hyperdense lesion in the right basal ganglia expanding the differential to include a subacute basal ganglia hemorrhage, vascular malformation, or a benign or malignant mass containing hemorrhages or calcifications. To further investigate this lesion, a CTA of his head and neck was completed that reported negative for any significant vascular disease. Further, an MRI of his head was completed and reported no territorial infarcts, but enhancement of the right caudate head and lentiform nucleus. The imaging allowed us to confidently exclude vascular diagnosis. With all images completed and the patient's presentation of unilateral chorea and non-ketotic hyperglycemia, the diagnosis of non-ketotic hyperglycemic hemichorea-hemiballismus syndrome could be determined. This diagnosis was likely due to hyperglycemia from his uncontrolled type 2 diabetes mellitus [1-3,9]. Glycemic control with a tailored insulin regimen was initiated to maintain euglycemia in preparation for discharge. With this diagnosis, our patients' emotional health and daily activities were significantly affected upon discharge. To maximize his quality of life and management of symptoms, tight glycemic control and physical therapy were continued outpatient. After two months of treatment, our patient showed notable improvement, with some moderate chorea still present and improving quality of life. In addition to the improvement of chorea symptoms, the tight glycemic control also allowed our patient's hemoglobin A1C to downtrend from greater than 15% to 8.4% in three months.

At this time, no neuroleptic has been prescribed to further manage his chorea. The decision to focus on glycemic control only can be supported by other case reports and studies listed above. Further, neuroleptics and benzodiazepines were not considered due to potential adverse effects and minimal long-term benefits. With this data and consideration, it can be presumed that he would benefit the most with strict glycemic control only [5,7]. He will continue to be monitored outpatient with expectations of complete resolution within six months [7].

## Conclusions

This case presents a rare complication of uncontrolled diabetes mellitus. Patients presenting with non-ketotic hyperglycemia and chorea should be evaluated with neuroimaging to evaluate for hyperintensity of the basal ganglia. With this diagnosis, prompt treatment of glycemic control should be initiated with additional consideration of neuroleptics for symptomatic control. Outpatient management with strict glycemic control should be maintained after resolution of symptoms to minimize the possibility of recurrence.
